# Kaplan-Meier Type Survival Curves for COVID-19: A Health Data Based Decision-Making Tool

**DOI:** 10.3389/fpubh.2021.646863

**Published:** 2021-10-25

**Authors:** J. M. Calabuig, L. M. García-Raffi, A. García-Valiente, E. A. Sánchez-Pérez

**Affiliations:** ^1^Instituto Universitario de Matemática Pura y Aplicada, Universitat Politècnica de València, Valencia, Spain; ^2^Universitat Pompeu i Fabra, Barcelona, Spain

**Keywords:** COVID-19, Kaplan-Meier, survival, decision, optimization

## Abstract

Countries are recording health information on the global spread of COVID-19 using different methods, sometimes changing the rules after a few days. All of them are publishing the number of new individuals infected, recovered and dead individuals, along with some supplementary material. These data are often recorded in a non-uniform manner and do not conform the standard definitions of these variables. In this paper we show that, using data from the first wave of the epidemic (February-June), Kaplan-Meier curves calculated with them could provide useful information on the dynamics of the disease in different countries. We developed our scheme based on the cumulative total number of infected, recovered and dead individuals provided by the countries. We present a robust and simple model to show certain characteristics of the evolution of the dynamic process, showing that the differences in evolution between countries are reflected in the corresponding Kaplan-Meier-type curves. We compare the curves obtained for the most affected countries at that time, with the corresponding interpretation of the properties that distinguish them. The model is revealed as a practical tool for countries in the management of the Healthcare System.

## 1. Introduction

Since its first detection in China, COVID 19—disease caused by SARS-CoV-2 virus—has spread to different parts of the world to reach pandemic status in a short period of time. This has created a social and scientific challenge, in which understanding how the virus behaves is crucial to stop its spread. A classic tool in the analysis of epidemics that could be used in this sense is the Kaplan-Meier (KM) survival model (1) that allows to calculate the stepwise survival probability of a fixed group of patients suffering from a disease [see for example (2, section 15) for a contextualised explanation of the topic]. Kaplan-Meier curves provide an easy and visual way to understand processes involving a population of individuals that is decreasing over time. They give the rate of individuals who still remain in a group—in our case, individuals who still remain under the control of the corresponding national health system—after a given time, and can therefore be understood as an estimate of the probability of this occurring. Comparisons of the curves for different countries provide a method for analyzing the different strategies that countries used at the onset of the pandemic. In a first approximation, the rapidly decreasing curves could be understood as a sign of the efficiency of the strategies developed by the countries, but this simplistic explanation cannot be established as a general rule, since the data presented by the countries on the pandemic also involve variables other than the response capacity of the health systems. However, we have found—and this is the main contribution of the present work. that countries can be grouped according to the shape of their Kaplan-Meier curves, which opens the door to a global analysis of the strategies—including data management, medical treatment, isolation measures and other relevant actions—used throughout the world. For this research, we collected data from the database of John Hopkins University (Coronavirus Resource Center), which provided an internationally accredited source of information. Of course, depending on the country the data were affected by a lot of different biases, so we have no choice but to assume that the national strategies we want to consider in our comparative analysis include the data management itself. In other words, we consider the management of these data as a component of the national strategies to fight Covid-19. But there is another fact that is even more problematic for the mathematical analysis: the data are aggregated—they are essentially given by the number of new infections, deaths and individuals cured each day, so a disaggregation (deconvolution) procedure is necessary to obtain Kaplan-Meier curves. The mathematical issue of doing this was studied in our work (3), where several analytical-heuristic methods are considered. Finally, we decided to use Genetic Algorithms for our overall analysis, as it was the method that was shown to be the most efficient. However, although the question of comparing different calculation procedures is interesting in itself from a mathematical and computational point of view, it is not the aim of the present work, in which we try to present some information on comparative treatments against pandemic situations using a synthetic information resource: Kaplan-Meier curves. The proposed method which would allow a prediction of how, given an average infected individual, his or her infection status changes over time. As we have explained in the precedent paragraph, the nature of data collected on the pandemic in different countries is diverse and this model needs to be adapted to the specific case of COVID-19 to provide relevant information. As will be shown in the paper, each country has its own survival curve with strong differences, which cannot be justified as a unique consequence of local population characteristics: a survival curve should only depend on the virus, assuming the usual degree of homogeneity in the infected population. Therefore, the reason for the strong difference in the results in different regions have to be sought in two directions: first, the way countries are reacting to the epidemic, and second, the characteristics of the data these countries have made public.

In addition, current models are not sensitive enough to capture the different dynamics of different virus strains. This is because viruses with RNA as their genetic material are less stable than those with DNA and tend to accumulate a greater number of mutations. This means that the virus changes more quickly, ending up in different variants of the same virus that have different mortality and infection rates. Furthermore this feature raises the fear about future cases of re-infection, where the virus differs enough from previous versions to evade the immune system again—as such other virus with the same genetic material do, e.g., Influenzavirus A, that causes the common flu and is able to infect us repeatedly (4). This scenario may occur as other coronaviruses are able to infect humans periodically such as HCoV-NL63 or HCoV-229, that are responsible of one out of five colds (5). The mutation rate for SARS-CoV-2 is not known yet, but given its potential, the possibility should be considered.

Thus, although all these arguments could influence the unusual results of the survival curves these facts do not substantially change the structure of the model (3). Therefore, the problem comes from the data. But this fact does not invalidate the usefulness of the Kaplan-Meier curves. Here, we show that some significant patterns can be detected by comparing the curves constructed for different countries. In further applications, survival curves could also provide some useful information for decision-making on the implementation of strategies against the spread of COVID-19, such as the length of confinement periods or the intensity of new case detection policies. In this work we use the available data of the dynamics of the disease COVID-19 to understand the survival of the virus that causes it, SARS-CoV-2. Although more information is already available on the second wave of COVID-19 in the countries we have analyzed, we have opted for the methodological approach of using data from the first wave—February to June. The reason is because this period defines a complete (almost closed) cycle of infection. Since we are interested in drawing some methodological conclusions from the experience, we believe that this procedure allows a more stable framework for obtaining them.

The results of our analysis are the estimates of the probability distributions of virus survival in different countries. These are functions that are sensitive to changes in the epidemiological data of different populations. This makes the model adaptable to reinfection scenarios and other more subtle differences such as the virulence of different strains (6). Together with some usual models for predicting the amount of new infected population, this allows the development of a complete model for the evolution of newly infected individuals, people who must be kept in quarantine and individuals who have already overcome the disease. To approximate the solution of the equations we use a genetic algorithm approach (7), which provides estimates of the probability and therefore clear images of the expected infection scenario. A full explanation of the mathematical method we have developed to do this is available in (3). In this paper we show that our model provides information that can be relevant for the management of health systems for different countries. We have found—and this is the main contribution of the present work. that countries can be grouped according to the shape of their Kaplan-Meier curves, which opens the door to a global analysis of the strategies—including data management, medical treatment, isolation measures and other relevant actions—used throughout the world. We believe that these results may allow us to monitor the effectiveness of containment policies, thus helping in the decision making process. The simplicity, both of the model itself and of its calculation and interpretation, is one of the main advantages of our approach, which makes it suitable as a forecasting tool.

Regarding other models being used in the pandemic crisis, a great effort is being made to improve the mathematical representation of the number of newly infected individuals in order to provide an accurate predictive tool. The most popular model being used is the SIR model and modifications of this model, that in particular provides a forecast of the number of new infected people in subsequent steps of the dynamical process [see for example (2, 8) and the references therein]. Also, other models have used time series to forecast the confirmed and recovered cases (9, 10).

However, the probability of survival of the virus could be even more relevant for the management of strategical information for decision-making regarding important data that affects the population in different countries. For example, to decide how long a period of confinement should last and to which type of population it should apply. The aim of this work is to define a general management and evaluation model based on Kaplan-Meier survival curves to assist healthcare system managers in their decision making. Other approaches has been done in the same sense for improving decision making using Machine Learning techniques (11). Our approach is similar to that presented in (12) but the mathematical setting is much easier and directly interpretable in terms of the system's ability to deal with the pandemic, as we will show in the next sections. This simplifies both its use and the data needed to feed the model. We would also like to point out that our model can also be used for resource planning for a particular hospital (13).

## 2. Methods

Our model is based on a modification of the classical Kaplan-Meier survival curve. The idea is to fit the evolution curve of the accumulated total amount of *recovered* (R) plus *dead* (D) people—data provided by countries—from the beginning of the epidemic to time *t*. We call this number X and we will refer it as *discharged people*. We consider X=R+D. On the basis of the accumulated total amount of *infected* people, (J), in the same period—data provided also by countries, the model fits J vs. the discharged ones (X) estimating the stepwise probability of the *virus to survive*—denoted by P—in a given infected patient. The result of our fit provides the time series of both the probabilities of survival of the virus, P, and the approximation $\hat{X}$ of the accumulated number of discharged people (X). We would like to point out here that other definitions of “people living with the virus”are possible: (i) people living with symptoms, (ii) infectious people, or (iii) people requiring health system care. All of these are useful for healthcare systems management, but due to the available data they cannot be used.

On the one hand, in the right panel of **Figures 2**–**4** it can be seen examples of the approximation of the function X for different countries. The black line corresponds to the real data of discharged people (X) while the red line is the result of our curve fitting ($\hat{X}$). As it can be seen, both curves are almost coincident for all the countries considered and in a period of time of 95 days. The reddish shaded area represents a range of 10% over the maximum value of X.

On the other hand, in the left panel of **Figures 2**–**4**, we show the representation of the survival curve of the virus. It gives the probability of an individual continuing to be infected—in terms of being under the control of the national healthcare system according to the data collection in each country—after the day when he/she was *labeled as infected* (which corresponds to *t* = 0 in the representation).

The Kaplan-Meier (KM) survival curve (1) is based on the estimation of the instantaneous probability of survival at a given time in the process of reduction of a given population. The interested reader can find a complete explanation of this and related topics in (14, Ch.2) and (15, 16). We assume that the time variable has discrete values. For the sake of simplicity of formulas and without loss of generality we will consider *t* ∈ ℕ starting at the moment *t* = 0. We write P(t) for the probability of an individual that has been labeled as “infected” is still infected by the virus at the time *t*. An estimate of this value for a given population of *N* infected individuals at the time *t* = 0 is given by

$$P\left(t\right)=\frac{n\left(t\right)}{N},\;t=0,1,2,3,\ldots ,$$

where *n*(*t*) is the number of patients that are still infected at the day *t*. Note that *n*(0) = *N* and P(0)=1.

Let us now turn our attention to the infection process that began in the first wave of the epidemic. Given a fixed country, let us write now *I*:ℕ → ℕ for the function that gives the number of new infected individuals *I*(*t*) at time *t*. The total amount $\hat{X}\left(t\right)$ of individuals surviving after a time *t* can be written as a KM type survival function given by the convolution formula

$$\hat{X}\left(t\right)=\overset{t}{\underset{s=0}{\sum }}I\left(s\right)\cdot (1-P\left(t-s\right)),\;t=1,2,3,\ldots ,$$

where P(u) is, as we have said, the probability that an individual is still infected at the time *u*. This quantity approximates the number of discharged people X(t). Using this equation it is possible to compute the Kaplan-Meier survival curve P from the data extracted of the reports of the different countries affected by the pandemia. The complete explanation of how this can be done can be found in (3).

## 3. Results and Discussion

**Figure 1:**
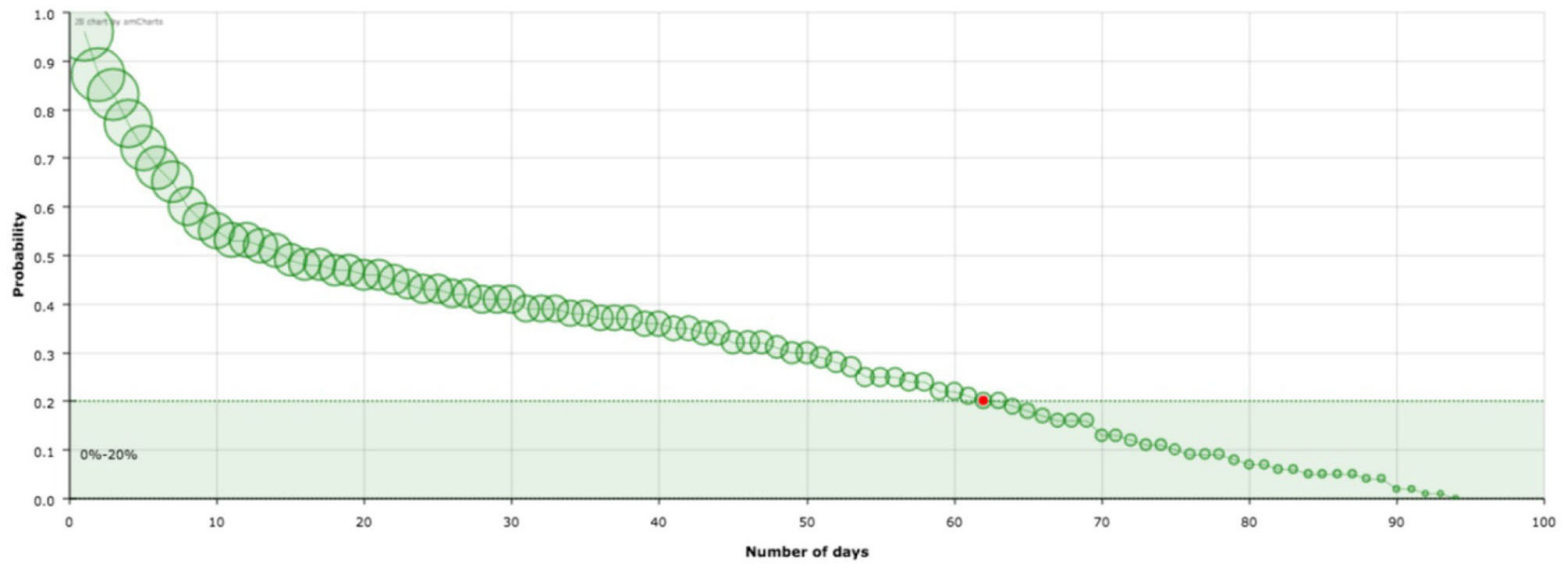
Survival curve, S, of the virus corresponding to Spain. The red point signs the number of days after which a standard individual has a probability of staying in the group of infected people smaller than 0.2. This value has been arbitrarily set as an aid to the visualization of the decreasing of the curve. The size of the balls is proportional to their value at the point.

In the previous section, we have introduce a model based on variables that are directly related to the epidemiological data provided by countries during the first wave of the Covid-19 pandemic. The meaning of the quantities appearing in this model has a straightforward interpretation. Let us start with the explanation of the probability distribution P. For example, in the case of Spain (Figure 1) 62 days after being classified as infected a standard patient has a probability of remaining infected of 0.2. In other words, 20% of the patients listed as infected the first day (*t* = 0) will still be labeled as infected after 62 days (remaining in the hospital or at home in quarantine). It can be seen that there is a significant decrease in the curve in the first 10 days. Indeed, since S(1)=0.96 then after one day 96% of infected people will still be infected whereas 10 days later only the 53% will be. However we need 52 days more to reduce the percentage to 20%. The shape of these curves is the main element of our analysis.

**Figure 2:**
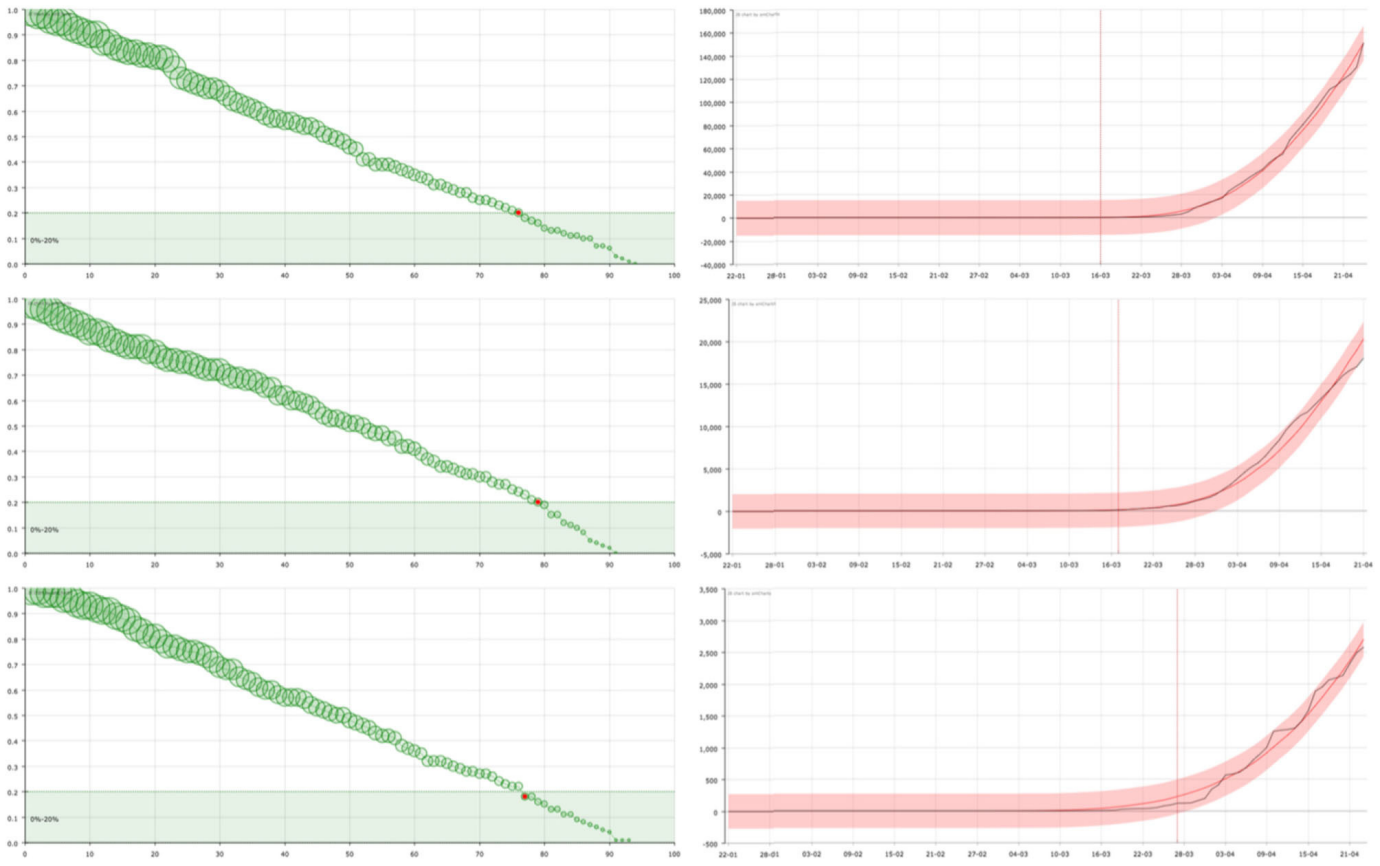
On the right, curve fitting of the sum of the accumulated number of recovered and dead people in USA **(top)**, United Kingdom **(center)**, and Sweden **(bottom)**: black line is the real value (X) and red line is the approximation ($\hat{X}$). The x-axis represent the date and the y-axis represents the number of cases. Vertical line signs the 100 cases. On the left, the corresponding survival curve of the virus S. The red point signs the number of days after which a standard individual has a probability of staying in the group of infected people smaller than 0.2. This value has been arbitrarily set as an aid to the visualization of the decreasing of the curve. The size of the balls is proportional to their value at the point.

**Figure 3:**
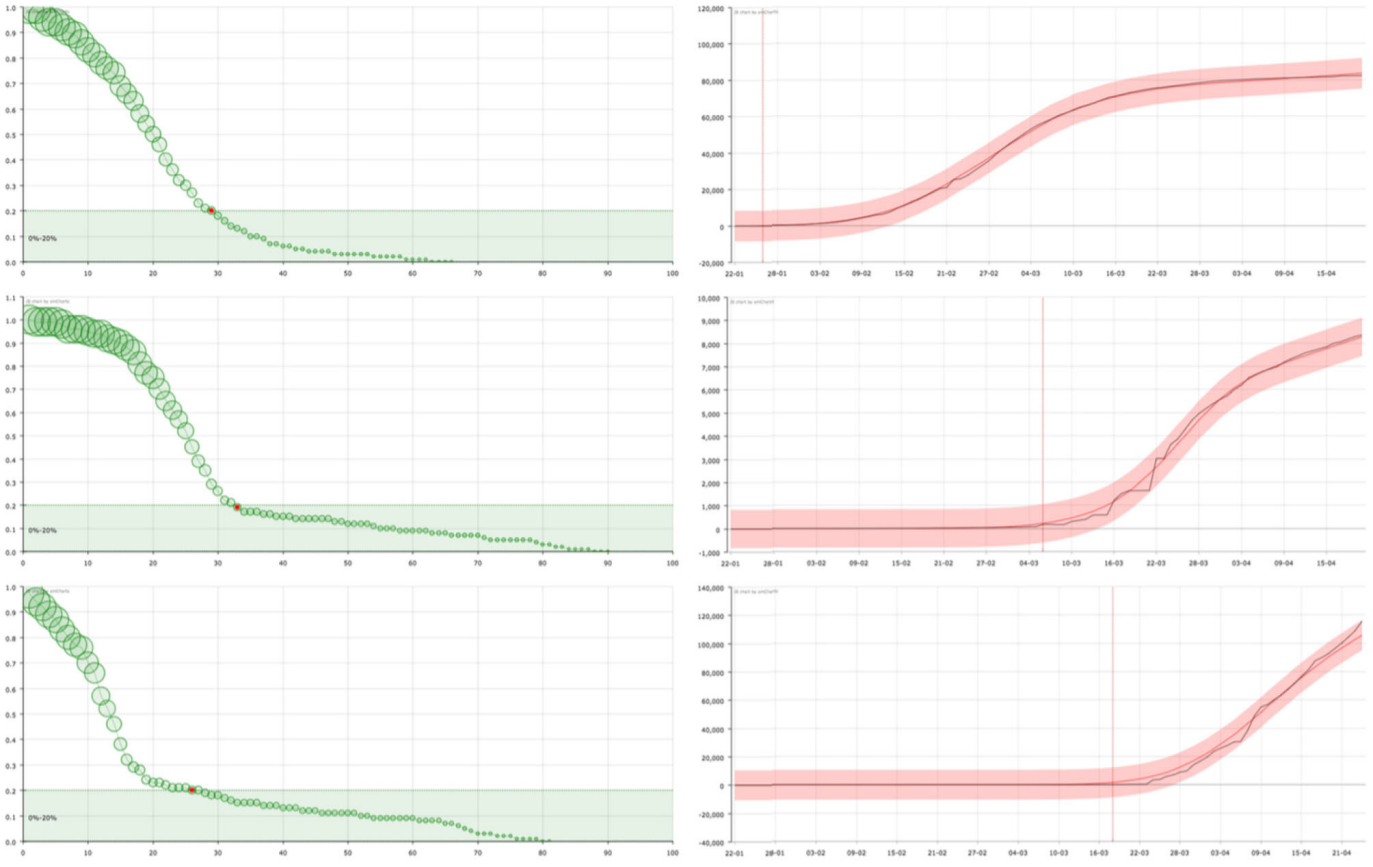
On the right, curve fitting of the sum of the accumulated number of recovered and dead people in China **(top)**, South Korea **(center)**, and Germany **(bottom)**: black line is the real value (X) and red line is the approximation ($\hat{X}$). The x-axis represent the date and the y-axis represents the number of cases. Vertical line signs the 100 cases. On the left, the corresponding survival curve of the virus S. The red point signs the number of days after which a standard individual has a probability of staying in the group of infected people smaller than 0.2. This value has been arbitrarily set as an aid to the visualization of the decreasing of the curve. The size of the balls is proportional to their value at the point.

**Figure 4:**
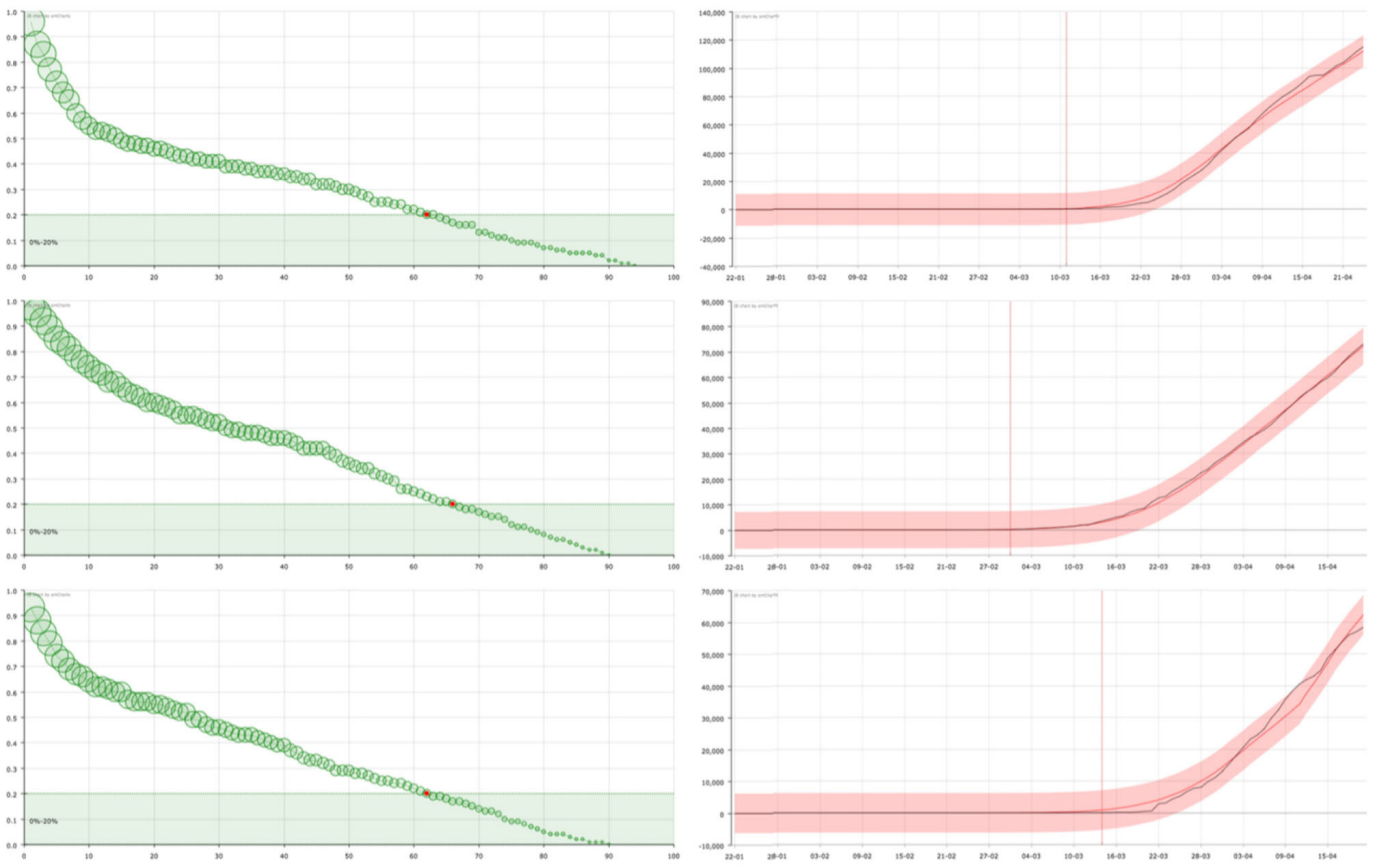
On the right, curve fitting of the sum of the accumulated number of recovered and dead people in Spain **(top)**, Italy **(center)**, and France **(bottom)**: black line is the real value (X) and red line is the approximation ($\hat{X}$). The x-axis represent the date and the y-axis represents the number of cases. Vertical line signs the 100 cases. On the left, the corresponding survival curve of the virus S. The red point signs the number of days after which a standard individual has a probability of staying in the group of infected people smaller than 0.2. This value has been arbitrarily set as an aid to the visualization of the decreasing of the curve. The size of the balls is proportional to their value at the point.

Here, the most remarkable feature is that, as can be seen in Figures 2–**4**, the model is sensitive to the progression of the epidemic in different countries, showing different patterns of survival curves.

In countries such as the United States or the United Kingdom, the form of the curves suggests that the spread of SARS-CoV-2 was not been effectively controlled at an early stage, either because no general testing of infected people was done or the government Health authorities decided to present the global numbers in a different way, not counting a big group of people suspicious of being infected. As a consequence, the reported number of “admissions” in the system (registered infected people J) is greater than the number of discharges (X) over a long period of time, so it takes longer to reach equilibrium. Then the individual's probability of getting out of the group of infected people decreases slowly (see Figure 2).

As opposed, there are countries where, after the first cases were detected, mobility was restricted and a large number of tests were carried out to identify and isolate infected persons—as the case of South Korea or Germany. The number of infections reported by these countries reveals this fact. The curves suggest that this policy was maintained throughout the whole process of the first wave of the epidemic. The number of “admissions,” although initially much higher, is rapidly decreasing, approaching the number of “discharges.” The curve shows a rapid decrease in the probability of an individual remaining infected, followed by a flattening of the curve in which a slower decrease is observed corresponding to the normal evolution of infected individuals in hospitals (Figure 3). This would also be caused by—or together to, a powerful campaigne of test made over all the general population, getting and reporting—as the figures provided by these countries reflect—a big number of individuals who were positive but asymptomatic, or who had a very good response to the medical treatment. In some cases such as in Korea, since the number of infected persons is not so large, the model shows the changes in trend with greater sensitivity. This allows us to see how the initial trend is similar to that observed in countries with late, deficient or ineffective control measures, with a strong decrease immediately afterwards.

Finally, in countries such as Spain or Italy (Figure 4), where the measures taken have partially slowed down the expansion, a less pronounced decline in the KM curve is observed, showing a mixed behavior between the two extreme cases that have been considered in Figures 2, 3.

Thus, the KM survival curve gives an estimate of the speed of the national system to detect and manage new infected individuals. A large number of tests makes it possible to control a relevant number of infected individuals (perhaps asymptomatic) reducing the stress for the national healthcare system because it can reduce the severity of the infections, i.e., the period in which infected individuals are under control of the healthcare system (with a lower use of clinical resources). This results in a considerable efficiency of the system, especially if done early in the epidemic, and (looking at the results) appears to be the most effective strategy. Early detection (at any stage of the process, but especially at the beginning) and massive testing, together with containment measures to reduce the rate of infection, appear to be the main weapons against the virus. Containment also appears to be an effective tool, but its effectiveness is based on other aspects of the system: it clearly reduces the number of new infections, but this may not affect the survival curve.

**Figure 5:**
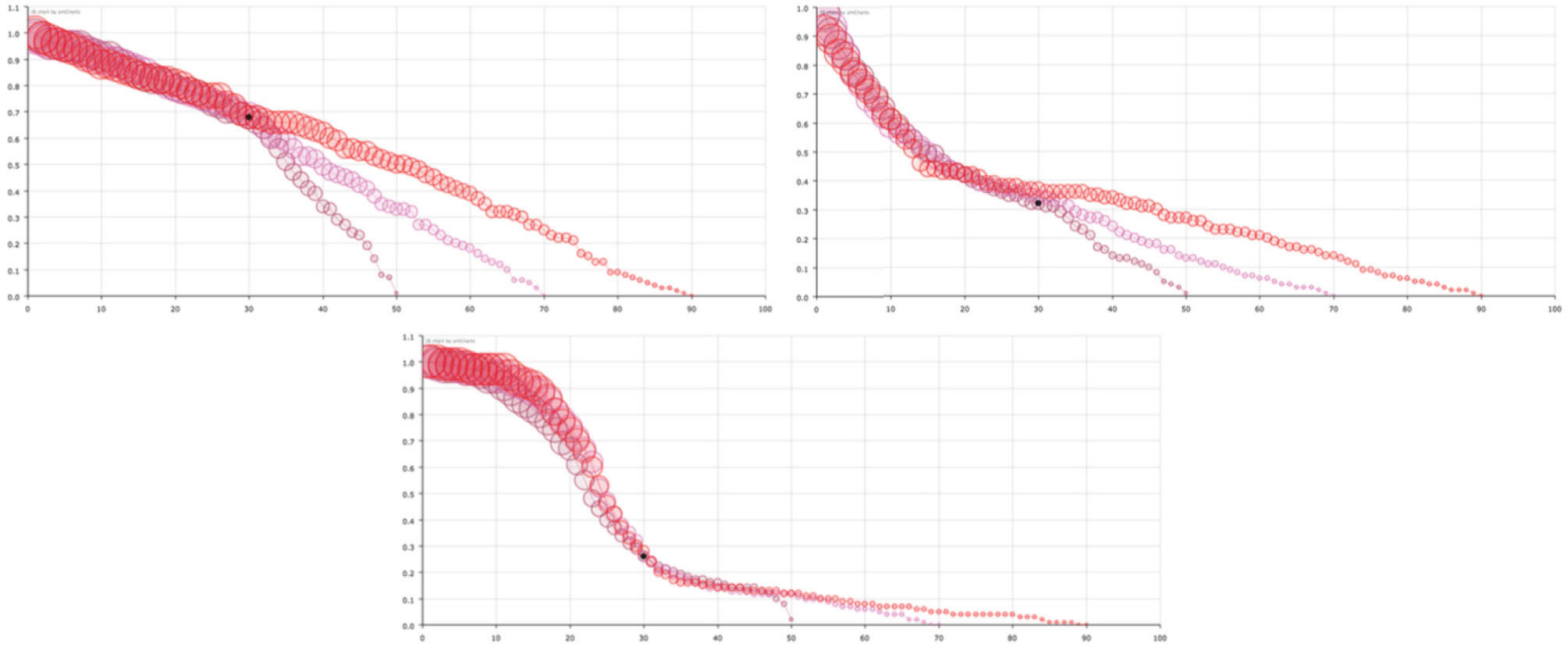
Survival curve of the virus considering different number of days for USA **(left-top)**, Spain **(right-top)**, and South Korea **(center-bottom)**. Red circles corresponds to data of the first 90 days, pink circles to only the first 70 days and maroon circles to only the first 50 days. The size of the balls is proportional to their value at the point.

In short, we can consider that the model can help the decision-makers of each country to know the distribution of time periods in which the healthcare system has to take care of infected people, according to the same variables that the healthcare policy makers have chosen to measure, in our case, infected (confirmed), recovered and dead people. Finally, in Figure 5, we show the variation of the survival curve of the virus, when computed with different time series of days (50, 70, and 90 days) that provide an idea of the stability of the solutions. Note that the principal feature of the curve, the decreasing in probability during the early period is maintained independent of the number of days considered.

The values of the slopes from the analysis of the tail of the survival curves are shown in Table 1. Countries that show a strong decrease after a few days, at the beginning of the curve, have lower slope values. This could be attributed to better medical treatment in countries with higher slopes, as the proportion of people leaving the system increases. However, as we assume that the medical methods used in all the countries analyzed are similar, this interpretation does not seem to be correct. Instead, it seems to be a consequence of the higher proportion of the population tested in the countries with small slopes: more people start to be followed by the healthcare systems at an early stage in countries such as South Korea or Germany, so the medical prognosis is statistically better. Therefore, of the total population followed in these countries, only a small rate needs medical attention in the late stage, but these patients need it for a long time, so the slope is small.

**Table 1:** Slopes of the linear final trends of the KM curves.

**USA**	**United Kingdom**	**Sweden**
−0.0106	−0.0102	−0.0109
**China**	**South Korea**	**Germany**
−0.0025	−0.003	−0.003
**France**	**Italy**	**Spain**
−0.008	−0.0087	−0.006

## 4. Conclusions

The aim of this work, as mentioned above, is to define a general model for the management and evaluation of healthcare systems based on the Kaplan-Meier survival curves of the virus. In particular, in this paper we use it to compare the efficiency of different healthcare systems (different countries) in dealing with the pandemic. In this particular case, our arguments can be summarized in relation to two different discursive axes:

1. If the data provided by all countries were comparable—that is, if the criteria for diagnosis, addition of new cases, deaths and cured patients were recorded according to the same rules—each country's Kaplan-Meier curve would accurately represent its capacity to manage the covid-19 crisis. Indeed:
  - An increased number of diagnostic tests registered for screening of new cases of infected persons results in a steep downward curve, as the health system detects more cases that are at an earlier stage of the infection, thus avoiding more serious complications and increasing the likelihood of a faster cure.
  - A faster action on the infected population results in a higher rate of patients being cured in a shorter period of time and therefore leaving the healthcare system earlier, reducing the stress of it.
2. We cannot assume that the data provided by countries are homogenous, i.e., the criteria for choosing indicators have not been the same everywhere. For example, the criteria for defining when a patient's death is caused by the virus have been different in each country. Therefore, the results presented in this paper are not definitive. However, and this is our main point, they are reasonable, as the comparison between countries and the groups defined are consistent with the general perception of which countries have had fewer problems in the first wave of the pandemic.

These arguments give a clear picture if the data taken by the different countries are comparable: the faster the Kaplan-Meier curve of the virus falls, the better the reaction of the health system to the crisis, and vice versa. Thus, the policies—special measures against the virus, closure of shops and stores, confinement of cities—adopted by countries with steepest downward curves tend to be better and this experience should be taken into account for future crises. It should be stressed that the total number of infected people is not the only parameter to monitor—experience shows that this variable is extraordinarily difficult to control—but that the capacity of healthcare systems to respond to the crisis must also be taken into account and is, in fact, the only relevant information for practical purposes.

There is also a technical problem caused by the fact that the data provided by public institutions are not disaggregated, i.e., they give an overall number of new infected people, deaths and cured per day but do not provide the number of days each patient is followed by the health services before being discharged and considered cured. This fact makes it necessary to deconvolute the data to obtain the survival curve using new mathematical procedures, as explained in (3), which inevitably introduce more errors. Analysis using complete data provided by the health administration would also reduce the error caused by this need for mathematical processing. This has been tested in (13).

Here, we have presented the estimates of virus surviving probability functions that have been calculated using the data available for the first wave in nine countries, which in a sense represent three different ways of data collection and healthcare system management. Epidemiology experts, data scientists and the public at large have noted that the count of newly infected, recovered and dead people depends on the country—the tools are not at all homogeneous—and does not reflect the real situation, mainly in terms of new cases of infected people. We have found the probability function for each country with the information made public by the corresponding governments during the first wave of the pandemic because it reflects the parameters that these same governments are able to measure and on which they can base their strategies.

The main characteristic of the curves is possibly the initial behavior, which allows us to group the nine countries we have selected into three categories. In our interpretation, this initial behavior reflects the way in which national healthcare systems are measuring the infected population as a whole: how many patients with some symptoms have been tested, and how they decide whether they should be under the supervision of the national healthcare system or not. Despite the known fact that the data provided by the countries are deficient, it is precisely the difference between countries in the shape of the curve that makes our model a useful tool from the point of view of epidemiology and healthcare system management. Under this assumption we have shown that the curve allows grouping countries according to the strategy followed to deal with the pandemic desease and in consequence it contains useful information about the different actions undertaken.

Regardless of how the variables are defined in each country—this has to be taken into account by the country itself when interpreting the results—the KM curve shows how quickly the healthcare system is able to deal with infected individuals: the faster the decrease of the KM curve in the first steps, the less pressure the system has to bear, since individuals need to spend less time controlled by this system. We emphasize that this control depends on how each country measures infection, and have to be understood in the context of each country. Different regions within a country could follow the same rules, and so could be compared.

However, some general conclusions can be drawn. The main one is that massive COVID-19 testing in the population improves the overall rating of the effectiveness of the healthcare system. This is clearly demonstrated by the survival curves in Germany and Korea, compared to other countries. As this can help control infected individuals, it allows countries to manage the healthcare system, resulting in a rapid decrease in the KM curve.

Finally, let us recall that the KM curve does not give a measure of how good the medical treatments are for the infected people in each country. Instead, once the counting method is fixed in each country, the KM curve provides decision-makers with a strategic tool for that country, as it gives a clear idea of how much time the healthcare system has to take care of an infected individual, whatever this means in the particular country's statistics. This could be relevant, for example, for the installation of emergency hospitals, the duration of special confinement measures, and other extraordinary measures.

## Data Availability Statement

The original contributions presented in the study are included in the article/Supplementary Material, further inquiries can be directed to the corresponding author/s.

## Author Contributions

JC, AG-V, LG-R, and ES-P designed the research and wrote the paper. JC, LG-R, and ES-P performed the research. All authors contributed to the article and approved the submitted version.

## Funding

The authors has been supported by the Cátedra de Transparencia y Gestión de Datos, Universitat Politècnica de València-Generalitat Valenciana.

## Conflict of Interest

The authors declare that the research was conducted in the absence of any commercial or financial relationships that could be construed as a potential conflict of interest.

## Publisher's Note

All claims expressed in this article are solely those of the authors and do not necessarily represent those of their affiliated organizations, or those of the publisher, the editors and the reviewers. Any product that may be evaluated in this article, or claim that may be made by its manufacturer, is not guaranteed or endorsed by the publisher.
