# Supplementary material for: Kaplan-Meier Type Survival Curves for COVID-19: A Health Data Based Decision-Making Tool
Source: Front Public Health. 2021 Oct 25;9:646863. doi: 10.3389/fpubh.2021.646863 (PMC8572972; doi:10.3389/fpubh.2021.646863)
Supplement: Supplementary file 2 [file Data_Sheet_2.PDF]

Supplementary Material

1 COMPUTATIONAL RESULTS

In this section we present the results of the paper concerning the computation of the Kaplan-Meier curves of the COVID-19 in several countries.To approximate the solution of the equations we use a genetic algorithm. This method belongs to the category of evolutionary algorithms (EAs), which mimic biological evolution. This is made possible thanks to the nature of GAs based on populations of individuals. We get profit on both the good results obtained with GAs, together with their capability to handle a wide variety of problems with different degrees of complexity, what explains their wide use (Yua and Gen, 2010). Regarding data, we have collected them from the Github of the "COVID-19 Data Repository by the Center for Systems Science and Engineering (CSSE) at Johns Hopkins University" accessible through `https://github.com/CSSEGISandData/COVID-19`. More concretely, we have made use of the confirmed, death and recovered global data from the time series available through the link `https://github.com/CSSEGISandData/COVID-19/tree/master/csse_covid_19_data/csse_covid_19_time_series`.

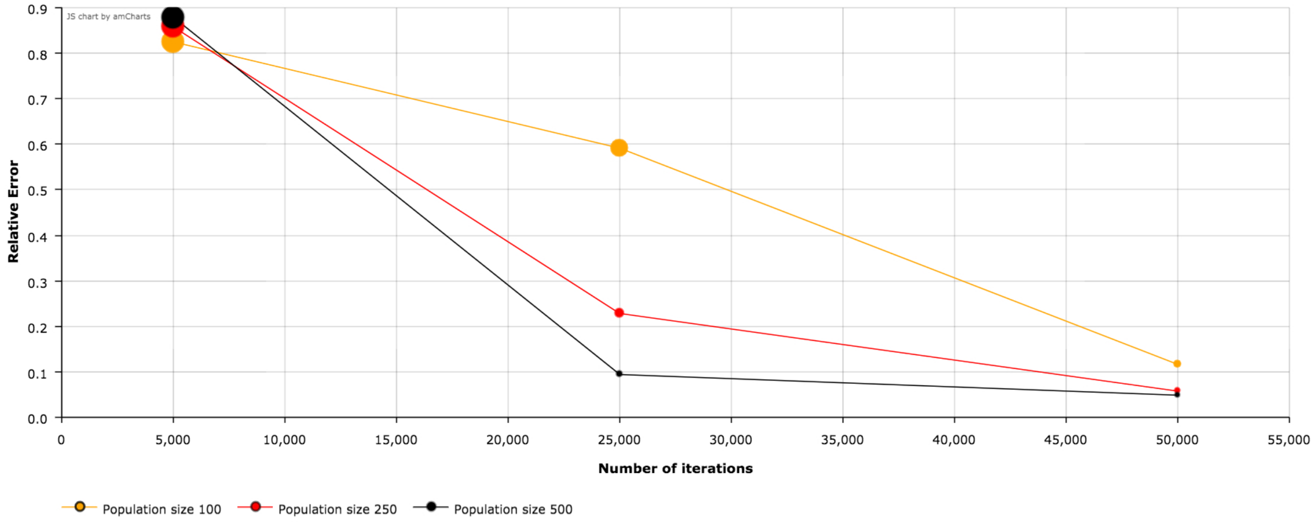

Figure S1. Variation of the relative error for the data corresponding to Spain in terms of `maxiter` for the three different values of `popSize`: pink (100), red (250) and black (500).

For doing that we have used the GA Package in R (Scrucca, 2013) defining a real-valued GA. In all the cases analyzed the convergence of the algorithm is very good. We have taken into account two arguments in the algorithm for the minimization of the error, the population size (`popSize`) and the number of iterations (`maxiter`). We have considered values of the `popSize` of 100, 250 and 500 in combination with `maxiter` that takes values equal to 5000, 25000 and 50000. In Figure S1 we have plotted for data from Spain the variation of the relative error in terms of `maxiter`. The orange, red and black lines correspond for `popSize`= 100, 250 and 500 sizes respectively. The plot for data from the rest of countries present the same behavior, selecting the values `maxiter`= 50000 and `popSize`= 250 for all the runs with an error lower than the 10%. Time execution of one instance (to fit the model to data from one country) in a Macbook2015 (Dual-Core Intel Core i5 2,7 GHz) with 8GB of memory laptop takes less than 30 minutes.

The results obtained by using this approximate method are good enough for our analysis. However, it has to be taken into account that the exact solution would give a slightly different picture of the survival distributions obtained. In (Calabuig et al., 2020) the reader can find a complete explanation.

2 SUPPLEMENTARY DATA

One of the most important factors for the reproducibility of research results is to make available to the scientific community the data that have been used to carry out the research work. The data used in this work has been obtained from `https://github.com/CSSEGISandData/COVID-19/tree/master/csse_covid_19_data` repository. In order to facilitate access to the data we have prepared several figures that appear in the paper in html format using the *rAmcharts* libraries in R . They are interactive figures that allow the researcher, simply by passing the cursor over them, to obtain a direct

reading of both the data that has been used as well as the values of the model presented. In addition the data can be downloaded in csv, xlsx or json format and also in png, jpg and svg format for the images (if desired, you can also select part of the images and download it), everything directly from the html. We believe that this can help to disseminate the content of our work, increasing its visibility and applicability.

REFERENCES

Yua X, Gen M. *Introduction to Evolutionary Algorithms* (Berlin: Springer-Verlag) (2010).

Scrucca L. Ga: A package for genetic algorithms in r. *Journal of Statistical Software* **53** (2013) 1–36. doi:10.18637/jss.v053.i04.

Calabuig JM, García-Raffi LM, García-Valiente A, Sánchez-Pérez EA. Evolution model for epidemic diseases based on the kaplan-meier curve determination. *Mathematics* **8** (2020) 1–25. doi:https://doi.org/10.3390/math8081260.
